# Robotic-Assisted Nissen Fundoplication in Pediatric Patients: A Matched Cohort Study

**DOI:** 10.3390/children11010112

**Published:** 2024-01-17

**Authors:** Rianne E. M. Killaars, Omar Mollema, Hamit Cakir, Ruben G. J. Visschers, Wim G. van Gemert

**Affiliations:** 1 Department of Pediatric Surgery, MosaKids Children’s Hospital, Maastricht University Medical Center+ (MUMC+), P. Debyelaan 25, 6229 HX Maastricht, The Netherlands; hamit.cakir@mumc.nl (H.C.); ruben.visschers@mumc.nl (R.G.J.V.);; 2 European Consortium of Pediatric Surgery (MUMC+, Uniklinik Aachen, Centre Hospitalier Chrétien Liège), Maastricht, P. Debyelaan 25, 6229 HX Maastricht, The Netherlands; 3 Research Institute of Nutrition and Translational Research in Metabolism (NUTRIM), Universiteitssingel 40, 6229 ER Maastricht, The Netherlands; 4 Faculty of Health, Medicine and Life Sciences (FHML), Maastricht University, Universiteitssingel 40, 6229 ER Maastricht, The Netherlands

**Keywords:** Nissen Fundoplication, robotic-assisted Nissen Fundoplication, pediatric patients, children, robotic-assisted surgery, senhance surgical system, Laparoscopic Nissen Fundoplication, gastroesophageal reflux disease

## Abstract

Background: Nissen Fundoplication (NF) is a frequently performed procedure in children. Robotic-assisted Nissen Fundoplication (RNF), with the utilization of the Senhance^®^ Surgical System (SSS^®^) (Asensus Surgical^®^ Inc., Durham, NC, USA) featuring 3 mm instruments, aims to improve precision and safety in pediatric surgery. This matched cohort study assesses the safety and feasibility of RNF in children using the SSS^®^, comparing it with Laparoscopic Nissen Fundoplication (LNF). Methods and Results: Twenty children underwent RNF with the SSS^®^ between 2020 to 2023 and were 1:1 matched with twenty LNF cases retrospectively selected from 2014 to 2023. Both groups were similar regarding male/female ratio, age, and weight. Two of the twenty RNF cases (10%) experienced intraoperative complications, whereas three in the LNF group of whom two required reinterventions. The observed percentage of postoperative complications was 5% in the RNF group compared to 15% in the LNF group (*p* = 0.625). The operative times in the RNF group significantly dropped towards the second study period (*p* = 0.024). Conclusions: Utilizing SSS^®^ for NF procedures in children is safe and feasible. Observational results may tentatively suggest that growing experiences and continued development will lead to better outcomes based on more accurate and safe surgery for children.

## 1. Introduction

Gastroesophageal reflux disease (GERD) is a common disease that affects about 23–40% of the pediatric population [1,2]. Individuals are suffering from long-lasting regurgitation of gastric contents into the esophagus causing symptoms such as epigastric pain, chronic cough, persistent vomiting, and failure to thrive. Clinical symptoms range from mild to severe. Especially, the pathological symptoms and complications including esophagitis and respiratory problems need to be avoided [1,3,4]. While GERD is primarily treated with diet, lifestyle treatment, and anti-reflux medication, symptoms can persist in some children despite conservative treatment. In severe intractable cases, anti-reflux surgery is necessary to alleviate symptoms and prevent complications [3,5,6]. 

Nissen Fundoplication (NF) is the most performed procedure in children suffering from GERD [7]. Based on fewer postoperative complications, less mortality, better cosmesis, and a shorter hospital stay, operating laparoscopically is offering a less invasive alternative to traditional open surgery [7,8]. However, especially in small children, standard laparoscopy still has limitations in terms of visualization and precision [7,8]. Robotic-Assisted Surgery (RAS) is emerging as a potential technique in pediatric surgery. The technique provides advantages such as more degrees of freedom, improved visualization, tremor filtration, implementation of augmented reality, and improved ergonomics and comfort for the surgeon [9,10,11]. Therefore, RAS allows for better operative precision, better cosmesis, and reduced trauma to surrounding tissue leading to less postoperative pain and a reduction in recovery time after surgery [9,10,11]. Specifically in GERD, there is a notable number of children who experience recurrent reflux after surgery [6,12,13,14]. The effectiveness of NF relies on precise and accurate technique [15]. Therefore, improving outcomes with the development of better and more precise operative techniques is expected.

Procedures in small children are challenging given the limited internal and external working spaces, changing bodily proportions, and more vulnerable tissues. RAS has the potential to overcome these obstacles. The Da Vinci^®^ Surgical System (DVS.S^®^) (Intuitive Surgical Inc., Sunnyvale, CA, USA) has already shown promising results in the pediatric population using instruments sized 5 and 8 mm [9,11,16,17,18]. However, the Senhance^®^ Surgical System (SSS^®^), with the utilization of 3 mm instruments, offers additional advantages, especially in small children and neonates [9,10,11,16,19].

The Department of Pediatric Surgery at the MosaKids Children’s Hospital of the Maastricht University Medical Center (MUMC+) performed the World’s first Robotic-assisted Nissen Fundoplication (RNF) by using the SSS^®^ in September 2020. To our knowledge, no report is present about RNF performed with SSS^®^ compared with LNF. The primary aim of this matched cohort study is to assess the safety and feasibility of RNF in children using the SSS^®^. Second, the study aims to compare RNF with LNF in terms of pre-, per-, and postoperative outcomes. 

## 2. Materials and Methods

### 2.1. Study Setting

This is a single-center prospective cohort study on RNF matched with a retrospective cohort of LNF. The study is performed at the MosaKids Children’s Hospital of the Maastricht University Medical Center (MUMC+), a Dutch academic hospital that serves as a referral and expertise center for the care of pediatric surgical patients. Children from 0 to 17 years, with a diagnosis of GERD who were operated on with the SSS^®^ from 2020 to 2023 were included in the study and followed in time. Informed consent was obtained from the parents and patients, or both depending on the patients’ age. 

### 2.2. Subjects

From September 2020 to November 2023, all consecutive pediatric patients with GERD, from 0 to 17 years, undergoing RNF with the SSS^®^ at the MosaKids Children’s Hospital, were included in the study. This cohort of children who underwent RNF was compared retrospectively with a 1:1 matched cohort of LNF cases. In both groups, GERD diagnosis and indication for surgery were made according to the Guideline of the European Society for Pediatric Gastroenterology, Hepatology, and Nutrition (ESPGHAN) [20]. The Senhance^®^ treatment guidelines were applied to all the RNF cases [21].

The matched cohort was extracted from a database, in which the medical records of all pediatric patients, aged 0 to 17 years, with GERD diagnosis and who underwent anti-reflux surgery in the MUMC+, were considered for enrollment. Of these, children who underwent LNF were included in the database. Patients who underwent an open procedure, a Toupet Fundoplication, a Collis-Nissen Fundoplication, or an LNF combined with another surgical procedure, were excluded from the database. Accordingly, the matched cohort of LNF was determined using an individual matching approach, pairing in a 1:1 ratio according to surgical procedure, age (within an accepted range of 2.5 years), and sex. The most recently performed LNFs were prioritized in consideration for enrollment. Following the introduction of the SSS^®^ in 2020 at MUMC+, RNF became the preferred method of surgery.

### 2.3. System and Surgical Team

The SSS^®^ consists of three sterile packed robotic arms and an unsterile open-platform surgical console with an ergonomic chair. Indications are approved in the US, EU, and Japan [21]. The robotic arms have several features, such as haptic feedback, digital fulcrum, and standard fully reusable instruments (3-mm, 5-mm, and 10-mm) similar to conventional laparoscopy. During surgery, 3 mm instruments were used in younger children while older children underwent procedures using 5 mm instruments. Furthermore, the surgical console offers 3D visualization with eye-tracking camera control.

All pediatric surgeons who performed the RNF procedures were surgical staff members and experienced with performing LNF. The surgeons and operating room assistants have been trained for the SSS^®^ in a dry and wet lab. With the introduction of the SSS^®^ at the Department of Pediatric Surgery at MUMC+ in September 2020, the first RNF was performed. During the study period from 2020 to 2023, RNFs were performed in a 1:4 ratio compared to other RAS procedures. In addition, a technical specialist from Asensus^®^ Surgical Inc. (Durham, NC, USA) has been present during the robot-assisted procedures to provide instructions and advice or to assist during robot malfunctions. 

### 2.4. Procedure

**Figure 1:**
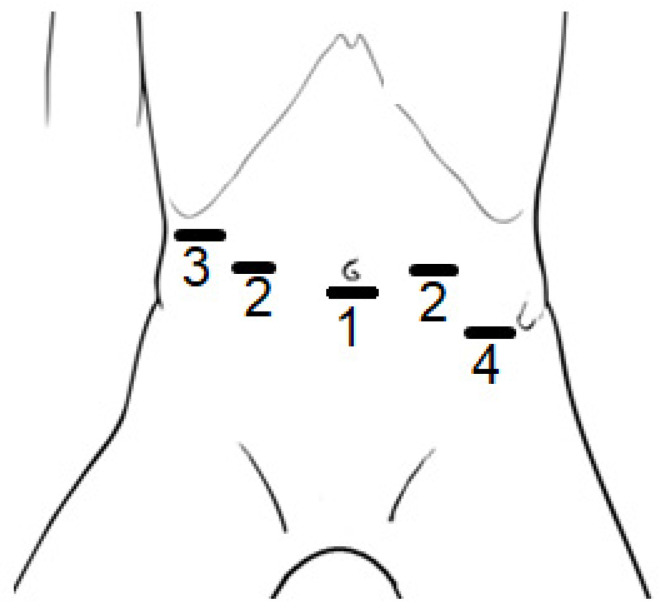
Positions of the incisions made for the trocars to be placed during Robotic-assisted Nissen Fundoplication (RNF) on children; (1) camera, (2) working trocars, (3) liver retractor, and (4) optional extra instrument. In larger patients, the position of incision 1 was supra-umbilical.

All patients were operated on in a supine position receiving general anesthesia, with the table in anti-Trendelenburg position. Figure 1 shows the anatomic locations of the incisions. Five trocars were inserted abdominally, of which three were occupied by the robotic arms (no. 1 and 2), and two were used for conventional laparoscopic instruments (no. 3 and no. 4). The camera was placed in position 1 (umbilical incision in smaller patients, and supra-umbilical incision in larger patients), and the two working trocars were in position 2. Position 3 was used for the liver retractor and position 4 (which is optional) was used as an extra instrument for the assistant or supervisor to give some additional help when needed. 

The RNF was similar to the LNF method. The procedures started with mobilization of the esophagus and gastric fundus as far as deemed necessary, and transection of the upper short gastric vessels in all cases. A retrogastric opening was created. Hiatal repair was performed using a single stitch (2-0/3-0 Mersilene, Ethicon, Johnson & Johnson, Raritan, NJ, USA). The construction of a floppy 360-degree NF was achieved with the placement of 2–3 sutures (2-0/3-0 Mersilene). 

### 2.5. Clinical Outcomes

Preoperative, intraoperative, and postoperative data from both the RNF and LNF groups were extracted from the patient’s records. During the robotic-assisted procedures, questionnaires were completed regarding the performance, perioperative course, and complications with the SSS^®^. As a result, collected data contained information on sex (male or female), age at the time of surgery, body weight at time of surgery, docking time of the robot (time from set-up till intra-operative use), operative time (time from incision to closure, in which docking time is included), conversion to conventional laparoscopy or open procedure, intraoperative complications, postoperative complications (within 30 days), classification of complications using Clavien–Dindo Classification [22], postoperative hospital stay (in nights spent following the operation), and readmission (within 30 days). 

### 2.6. Statistical Analysis

Descriptive statistics are used for the general presentation of patient demographics. Data analysis focused on comparing outcome measures between the RNF and LNF groups. All variables were assessed for normal distribution with the Shapiro–Wilk’s test and visual inspection of their histograms or Normal Q-Q Plots. To compare both operation techniques a paired *t*-test or Wilcoxon signed ranked test was performed for the continuous variables, where appropriate. Nominal data were assessed using the McNemar test. A paired *t*-test was performed to determine whether there is a statistically significant difference in mean operative time between the first and the second time period of the study. A boxplot and trend graph was performed to give an overview of the operative times over time. Corresponding effect sizes (mean difference, 95%-confidence intervals, and odds ratio) were calculated to assess clinical relevance. A *p*-value < 0.05 was considered statistically significant. The statistical analysis was performed using IBM Statistical Product and Service Solutions (SPSS) Statistics 28.0.

## 3. Results

### 3.1. Patient Characteristics

Between September 2020 and November 2023, a total of twenty consecutive pediatric patients underwent RNF using the SSS^®^ and were included in the study. All included patients had symptomatic GERD, confirmed by additional diagnostics, and gave written consent to be operated on with the SSS^®^. 

Among the children in the database, 133 underwent LNF. From this cohort, twenty LNF cases were individually matched in a 1:1 ratio with RNF cases, selected from the years 2012 to 2023, based on age, and sex. Both groups consisted of eleven men and nine women. The ages of the RNF group ranged from 0.8 years to 17.8 years old. The mean age in the RNF group was 7.9 ± 6.0 years, compared to 8.3 ± 6.1 years (age range 1.0–18.1) in the LNF group (*p* = 0.048). The mean body weight was 30.3 ± 20.3 kg (range 9.6–68.0) in the RNF group and 26.7 ± 17.9 kg (range 5.5–64.0) in the LNF group.

In the twenty children operated on using the SSS^®^, 3 mm instruments were used in eight cases (Table 1 and Table 2).

### 3.2. Peroperative Outcomes

All study data were successfully collected for all the cases, but in two the docking time was missing. Each RNF procedure involved a team of two pediatric surgeons: one operated behind the surgical console, and the other assumed a sterile position at the surgical table. In total, three surgeons alternately participated throughout the procedures. 

The mean operation time of the RNF procedures was 142 ± 38 min (range 87–247) with an included mean docking time of the robot of 7 ± 5 min (range 1–22, *n* = 18). The learning curve, regarding optimal setup of the robot, which varies based on child’s size, contributes to a significantly longer mean operative time for RNF compared to LNF procedures (142 ± 38 vs. 93 ± 33 min, *p* ≤ 0.001). 

**Figure 2:**
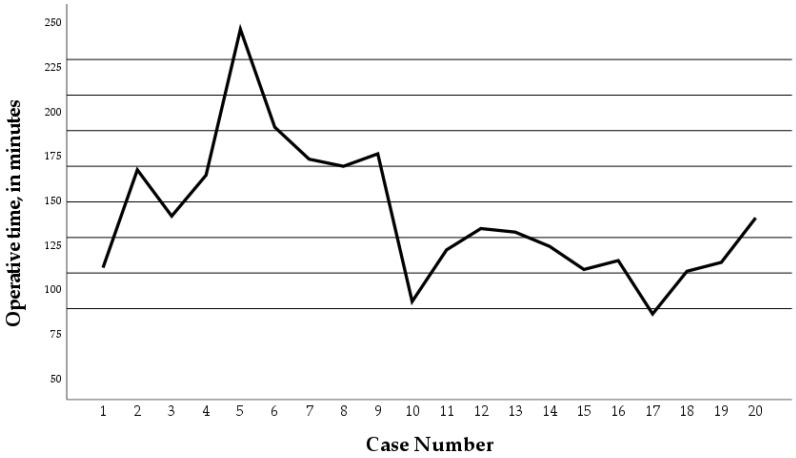
Operative times of the twenty Robotic Nissen Fundoplication (RNF) cases. Operative time dropped during the second time period of the study. Case number 5 and 8 converted to conventional laparoscopy.

**Figure 3:**
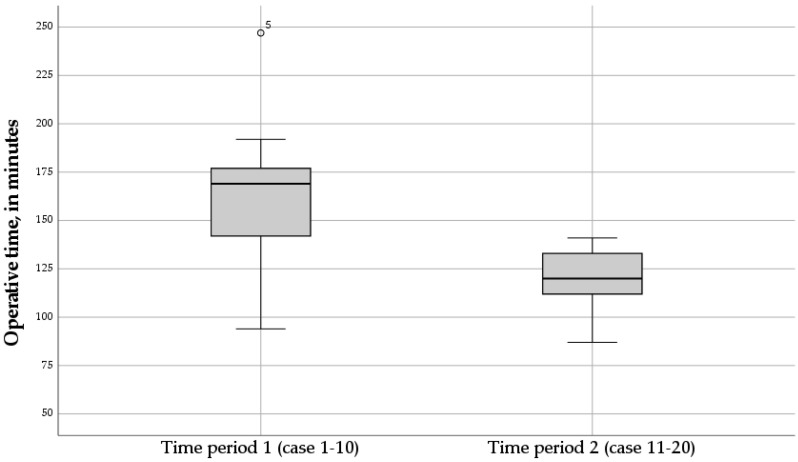
Boxplot of the operative times of the twenty Robotic Nissen Fundoplication (RNF) cases, for time period 1 (case 1–10), and time period 2 (case 11–20). The mean operative time in the second time period of the study was significantly lower compared to the first time period of the study (*p* = 0.024).

Over time, the trend of the operative time in the RNF group dropped during the second time period of the study (Figure 2). The mean operative time in the second time period of the study (case 11–20) was significantly lower compared to the first time period of the study (case 1–10) (*p* = 0.024) (Figure 3). 

In two of twenty cases (10%), RNF was converted to conventional laparoscopy, being case 5 and 8 during the first time period of the study. This was caused by a defective instrument in one patient. The other was converted to enhance visualization when iatrogenic damage to the esophagus was suspected. After inspection, there was only a small serosal lesion without perforation. Preventive suturing was performed, along with additional postoperative antibiotics, and was therefore classified as a Clavien Grade 2 complication. 

None of RNF procedures converted to an open procedure, whereas in the LNF group conversion to open surgery occurred twice because of an inadequate view (0% vs. 10%).

In total, two of nineteen RNF cases experienced intraoperative complications (10%). There was one Clavien Grade 4 complication, involving laparoscopic exploration on postoperative day three for a thermic gastric perforation. In the LNF group, the total number of surgical complications was three (15%) of which two patients required reintervention. One LNF-child required three reinterventions because of iatrogenic damage of the anterior vagus nerve, abscess drainage, and perforation of the esophagus (Clavien Grade 4). The second case of reintervention involved a nasoduodenal tube sutured in the wrap, requiring gastroscopic cutting of the suture (Clavien Grade 3b). The third case included a gastric perforation that was sutured intraoperatively (Clavien Grade 2) (Table 2).

### 3.3. Postoperative Outcomes

Observed percentage of postoperative complications within 30 days was 5% in the RNF group compared to 15% in the LNF group (*p* = 0.625). The mean duration of hospital stay was 3.3 ± 2.0 days in the RNF group, and 5.9 ± 7.5 days in the LNF group (*p* = 0.154). In both groups, one child suffered from passage problems through the Nissen wrap requiring endoscopic dilatation (Clavien Grade 3b). Only in the LNF group, there were an additional two cases with a wound hematoma opened at bedside, and a superficial wound infection (both Clavien Grade 1). There were no deaths in either group (Table 2).

Overall, 85% of the RNF cases were successfully completed without any complications compared to 70% of the LNF cases. 

## 4. Discussion

To the best of our knowledge, this is the first cohort study of RNF using the SSS^®^ in the pediatric population. The findings of the study provide new information about the use of SSS^®^ being safe and feasible. Additionally, the success rate and number of complications do not differ from conventional laparoscopy when comparing clinical outcomes. 

Several robotic procedures of NF have already been described in the pediatric population. The first case report of a performed NF in a child using the DVS.S^®^ (Intuitive Surgical Inc., Sunnyvale, CA, USA) was described in 2001 [23,24]. At present, a few studies compared RNF with the standard laparoscopic approach to Nissen fundoplication in children [18,25,26]. However, most of these studies have focused on the operative time and costs as important variables for comparison, or all the reported fundoplications were performed using the DVS.S^®^.

The SSS^®^ with digital laparoscopy focuses on 3 mm instruments for procedures in small children. RAS has many potential advantages compared to conventional laparoscopies, such as motion scaling, tremor filtration, and elimination of the fulcrum effect which makes it suitable for pediatric procedures [19]. In pediatric surgery, laparoscopic procedures in small children and newborns are mostly performed using 3 mm instruments. Furthermore, trocar positions need to be adjusted according to age, body size, and proportions. The SSS^®^ offers the possibility of using 3 mm instruments, which opens the door to RAS in small children and newborns.

The findings of the present study show a very acceptable success- and complication rate of the RNF procedures with the use of SSS^®^, as compared to reported results in previous literature [6,19,25,26]. In this study, the comparison of twenty children who underwent RNF with twenty children who underwent LNF showed that surgical- and postoperative complication rates were not different in both groups, demonstrating that the RNF approach is not inferior to the LNF. 

NF is commonly performed in children suffering from GERD; however, the procedure is inherently associated with a certain complication rate [6,27]. Meehan et al. described an overall complication rate of 14% in 50 children who underwent a RNF with the use of the DVS.S^®^ [28]. In this study, the observed percentage of postoperative complications was lower in the RNF group compared to the LNF group with a shorter duration of hospital stay, although both did not reach statistical significance. Results not reaching statistical significance might be the consequence of the small study population, however, it seems more plausible that there is currently no actual clinical difference in outcomes. Nevertheless, experience leads us to believe that growing experiences and continued development will lead to better outcomes based on more accurate and safe surgery for children. To study on whether RNF might be superior to LNF, further prospective studies with larger sample sizes are needed to expand the evidence and provide further information. 

The overall conversion rate from RNF to conventional laparoscopy is low (2/19, 10%), noting the conversions were due to a defective instrument and a precaution for patients’ safety. The acceptable conversion rate for pediatric populations has been reported to be 2.5–12% [29,30]. 

The absence of conversions from RNF to an open procedure indicates the robotic-assisted approach to be feasible. In contrast to the two conversions to open procedures in the LNF group, this might underscore the potential advantages of RNF in terms of maintaining a minimally invasive approach. This seems especially true in a modular system such as the SSS^®^ system which supports more flexibility in trocar positioning similar to the conventional laparoscopic approach and therefore facilitating conversion to laparoscopy. 

The mean operative time for RNF procedures was significantly longer compared to LNF procedures. Although literature reports differently on the association between robotic-assisted pediatric surgery and longer operation time, this can be explained by the learning curve of the robot [9,17,23]. The additional operative time in the RNF group was mainly attributed to optimizing the robot setup going along with factors like positioning the patient, trocar placements and positions of the robotic arms. Another important factor to reckon with is the very different sizes and proportions of children of different ages. This must be considered when setting up the SSS^®^ system and it takes some experience to do this correctly. Robotic malfunctions and requirement of conversion to laparoscopy have also impacted operative time. Notably, over time, the mean operative time for RNF cases in the second time period of the study was significantly lower compared to the first time period of the study, indicating an improvement in efficiency as the operative team became more familiar with the robotic approach. It is anticipated that the surgeons were still in the learning phase by the time this study ended, as indicated by the curve of the operative times over time showing a downward trend but has not yet reached a plateau phase. By contrast, surgeons are already familiar with performing conventional laparoscopy, which results in being easy to implement and faster acceptance of this robotic-assisted laparoscopic technique in clinical practice.

While the study primarily focused on the safety and feasibility of RNF using the SSS^®^, comparing outcomes with conventional laparoscopy remains a limitation of the study. The sample size of the paired groups is relatively small, limiting the strength of making any statements regarding outcomes of the study. The time period during which RNFs were performed differs from the time period for LNFs, leading to a variation in the surgeons who performed the procedures within the two study groups. Considering its extended period of time, the LNF group may involve a larger number of different surgeons. The relatively small patient number involved in this study implies that the comparison involves the learning curve of RNF against the last years of experience in LNF.

Finally, the SSS^®^ offers a comfortable ergonomic set-up. The position of the chair is adjustable according to the procedure and posture of the surgeon. Furthermore, especially in RNF, conventional trocar positions (anatomic locations used during conventional laparoscopy) can easily be maintained. However, it is critical to have accurate patient positioning and adequate trocar placement because of the limited external and internal working space in children. 

## 5. Conclusions

The current study demonstrates that the use of SSS^®^ for RNF procedures in children is safe and feasible. Some specific considerations must be taken into account when performing RAS in small children. Implementation in clinical practice and further development of the SSS^®^ system based on user experience is essential to optimize the system. We are confident that ongoing technological development in combination with increased experience will lead to better outcome based on more accurate and safe surgery for children.

## Figures and Tables

**Table 1:** Patient demographics for Robotic-assisted Nissen Fundoplication (RNF) patients.

P	Age at Surgery ^1^	Weight ^2^	Sex ^3^	Instruments Size ^4^
1	17.1	68.0	F	5 mm
2	12.2	38.0	M	5 mm
3	17.8	58.8	M	5 mm
4	1.5	9.6	F	3 mm
5	1.8	12.2	M	3 mm
6	0.8	10.1	F	3 mm
7	14.1	49	M	5 mm
8	0.9	10.3	M	3 mm
9	10.4	45	M	5 mm
10	15.8	65	M	5 mm
11	12.3	32	F	3 mm
12	3.2	13	F	3 mm
13	3.8	18	M	5 mm
14	3.7	16.5	M	3 mm
15	5.1	17.3	F	5 mm
16	3.6	15.6	M	5 mm
17	14.1	55	F	5 mm
18	1.9	10.5	M	3 mm
19	7.8	22.4	F	5 mm
20	10.1	40.3	F	5 mm

^1^ Age at time of surgery, in years. ^2^ Body weight at time of surgery, in kilograms. ^3^ F = female, M = male. ^4^ Size of the (work) instruments of the Senhance^®^ Surgical System used during the procedure.

**Table 2:** Clinical characteristics and outcomes of pediatric patients with gastro-esophageal reflux disease (GERD) who underwent either a Robotic-assisted Nissen Fundoplication (RNF) using the Senhance^®^ Surgical System (*n* = 20) or a Laparoscopic Nissen Fundoplication (LNF) with Conventional Laparoscopy (*n* = 20).

	RNF (n = 20)	LNF (n = 20)	Mean Difference (95% CI)	Odds Ratio (95% CI)	*p* Value ^1^
**Clinical characteristics**					
Sex in no. (%)					1.000
Male	11 (55)	11 (55)			
Female	9 (45)	9 (45)			
Age at time of surgery, mean in y (SD)	7.9 (±6.0)	8.3 (±6.1)	−0.31 (−0.64–0.01)		0.048
Body weight at time of surgery, mean in kg (SD)	30.3 (±20.3)	26.7 (±17.9)	3.66 (−2.04–9.35)		0.030
**Intraoperative**					
Conversion to conventional laparoscopy, no. (%)	2 (10)	-			
Conversion to open procedure, no. (%)	0 (0)	2 (10)			0.500
Operative time ^2^, mean in minutes (SD)		93 (±33)	50 (27–72)		<0.001
Total study time	142 (±38)				
Period 1 (case 1–10)	164 (±42)				
Period 2 (case 11–20)	120 (±15)		44 (7–81)		0.024
Docking time of robot ^3^, mean in minutes (SD)	7 (±5)	-			
Peroperative complications, no. (%)	2 (10)	3 (15)		1.5 (0.25–8.98)	1.000
**Postoperative**					
Postoperative hospital stays ^4^, mean in days (SD)	3.3 (±2.0)	5.9 (±7.5)	−2.65 (−6.15–0.85)		0.154
Postoperative complications ^5^, no. (%)	1 (5)	3 (15)		3.0 (0.31–28.84)	0.625
Readmission through 30 days, no. (%)	2 (10)	0 (0)			0.500
Reintervention, no. (%)	2 (10)	3 (15)		2.0 (0.18–22.06)	1.000
Mortality, no. (%)	0 (0)	0 (0)			
**Clavien–Dindo Classification for complications [22], no.**					
1	0	2			
2	1	1			
3a	0	0			
3b	1	2			
4a	1	1			
4b	0	0			
5	0	0			
Total number of complications, no. (%)	3 (15)	5 (30)			

^1^ Paired *t*-test or Wilcoxon signed ranked test was performed for the continuous variables, where appropriate. Nominal data were assessed using the McNemar test. *p*-value < 0.05 is considered as the level of statistical significance. ^2^ Operative time is the time from first skin incision to last wound closure. ^3^ Docking time is the time to put the robot in position with the instruments in correct position. The mean docking time was calculated based on the data available for 18 patients. ^4^ Postoperative hospital stay is defined as nights spent following the operation (i.e., discharge on the same day counts as 0, discharge the following day counts as 1). ^5^ Postoperative complications are complications arising postoperatively within 30 days.

## Data Availability

All data are stored securely at the Department of Pediatric Surgery of MosaKids Children’s Hospital/Maastricht University Medical Center+ (MUMC+) without patient identifiers and is available for inspection upon request.
